# Diagnosis and follow-up of posterior inferior cerebellar artery dissection complicated with ischemic stroke assisted by T1-VISTA: a report of two cases

**DOI:** 10.1186/s12883-016-0637-9

**Published:** 2016-07-29

**Authors:** Koji Ishitsuka, Yusuke Sakaki, Shota Sakai, Takeshi Uwatoko, Hitoshi Aibe, Tetsuro Ago, Takanari Kitazono, Hiroshi Sugimori

**Affiliations:** 1 Department of Cerebrovascular Medicine, Saga Medical Center Koseikan, Kase-machi Nakabaru 400, Saga, 840-8571 Japan; 2 Department of Cerebrovascular Disease, Japanese Fukuoka Red Cross Hospital, Fukuoka, 815-8555 Japan; 3 Department of Radiology, Saga Medical Center Koseikan, Saga, 840-8571 Japan; 4 Department of Medicine and Clinical Science, Graduate School of Medical Sciences, Kyushu University, Fukuoka, 812-8582 Japan

**Keywords:** MRI, PICA dissection, Intracranial

## Abstract

**Background:**

Volume isotropic turbo spin-echo acquisition (VISTA) is a new method similar to the 3D black-blood imaging method that enables visualization of a intramural hematoma. T1-VISTA has recently been applied in the diagnosis of intracranial arterial dissection. However, the identification of an intramural hematoma in posterior inferior cerebellar dissection (PICA-D) by T1-VISTA has only rarely been reported.

**Case presentation:**

We herein report two patients who suffered from PICA-D complicated with ischemic stroke. Initial magnetic resonance arteriography was not informative, however, T1-VISTA depicted high-intensity signal areas suggesting an intramural hematoma of PICA-D in both cases. The high-intensity signal areas gradually reduced and finally disappeared at 4 months and 5 months after the onset, respectively.

**Conclusion:**

Our cases demonstrate that T1-VISTA was able to assist in the diagnosis and follow-up of PICA-D.

## Background

Intracranial arterial dissection is an important cause of ischemic stroke, especially in young and middle-aged patients [1]. While vertebral artery dissection (VA-D) is the most frequent among intracranial arterial dissections complicated with ischemic stroke, posterior inferior cerebellar dissection (PICA-D) is rare [2]. Volume isotropic turbo spin-echo acquisition (VISTA) is a new method similar to the 3D black-blood imaging (BBI) method that enables visualization of a mural hematoma and has been applied in the diagnosis of intracranial arterial dissection [3]. Only one paper previously reported the identification of an intramural hematoma of PICA-D using T1-VISTA [3], whereas no study has been available for its follow-up. In the present report, we describe that T1-VISTA was helpful for the diagnosis and follow-up in our two cases of PICA-D complicated with ischemic stroke.

## Case presentation

### Case 1

**Fig. 1:**
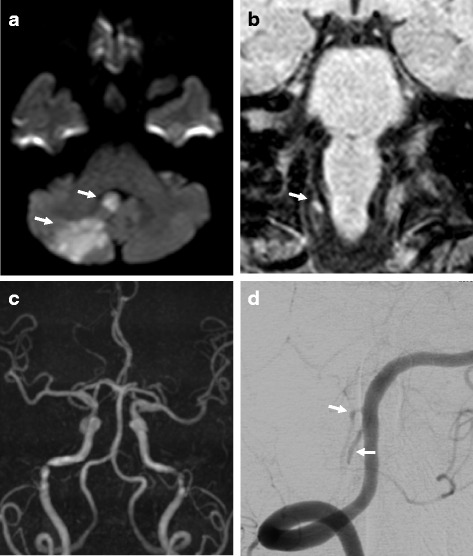
Imaging findings in Case 1. MRI on admission showed high-intensity signal areas in the cerebellar vermis and the right cerebellar hemisphere on DWI (**a**, arrow) and a high-intensity signal area beside the right VA on T1-VISTA (**b**, arrow). The right PICA was not detected on MRA (**c**). A right vertebral angiogram at 9 days after admission showed the “pearl and string sign” at the proximal portion of the right PICA (**d**, arrow)

**Fig. 2:**
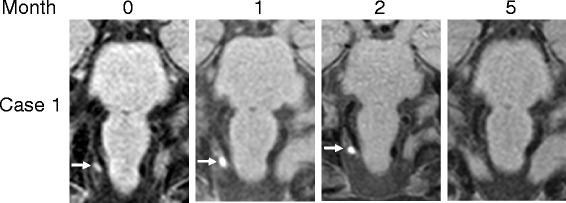
T1-VISTA imaging Case 1. High-intensity signal areas gradually resolved and finally disappeared

A 56-year-old woman began to suffer from an occipital headache for 7 days before admission to our hospital, and dizziness appeared 5 days later. On admission, she complained of dizziness and a mild occipital headache. She showed nystagmus and the right nose-finger-nose test was positive. In peripheral blood cell counts and biochemical studies, elevations in white blood cells (9,200/μl) and low-density lipoprotein cholesterol (215 mg/dl) were observed. Coagulation studies and immunological studies were normal. Head computed tomography (CT) showed low-density areas in the right cerebellar hemisphere, diffusion weighted imaging (DWI) of magnetic resonance imaging (MRI) displayed high-intensity signal areas in the cerebellar vermis and the right cerebellar hemisphere (Fig. 1a). In addition, a high-intensity signal area was observed just adjacent to the right VA on T1-VISTA (Fig. 1b), whereas no such high intensity was detected on conventional T1 image. Conventional MRA did not depict the right PICA or any abnormal findings of the right VA (Fig. 1c). Echocardiography and 24-hour electrocardiography showed no findings suggestive of cardioembolic causes. According to these findings, ischemic stroke of the cerebellum induced by the right PICA-D was diagnosed, and after confirming that she had neither hypertension nor aneurysmal formation, combined therapy with clopidogrel and unfractionated heparin administration was started. T1-VISTA showed a slight expansion of the high-intensity area at 3 days after admission, but still no lesions on T1 images. At 9 days after admission, cerebral angiography revealed the “pearl and string sign” at the proximal portion of the right PICA (Fig. 1d). At 12 days after admission, the patient was discharged with single antiplatelet therapy (clopidogrel). On T1 images, a pale high-intensity area was documented at the dissected lesion at 19 days after admission. Thereafter, the right PICA was gradually depicted on MRA and completely visualized at 5 months after the onset. In parallel with that, the high-intensity signal area on T1-VISTA gradually decreased in size and disappeared by 5 months after the onset (Fig. 2). Clopidogrel administration was then stopped.

### Case 2

**Fig. 3:**
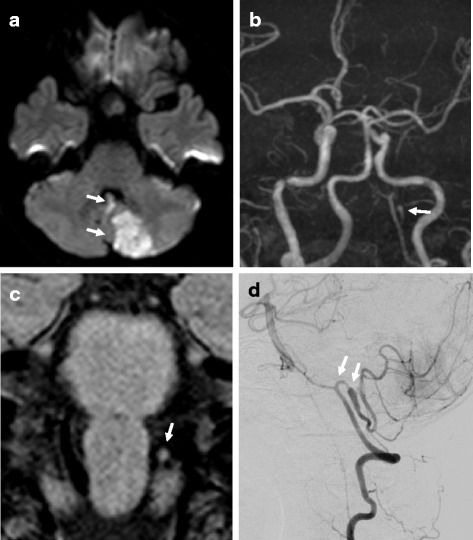
Imaging findings in Case 2. MRI at 4 days after admission showed high-intensity signal areas in the cerebellar vermis and the left cerebellar hemisphere on DWI (**a**, arrow), the loss of the proximal portion of the left PICA and dilatation of the adjacent distal portion on MRA (**b**, arrow), and a high-intensity signal area at the dilatation portion on T1-VISTA (**c**, arrow). A left vertebral angiogram at 8 days after admission showed the “pearl and string sign” at the proximal portion of the left PICA (**d**, arrow)

**Fig. 4:**
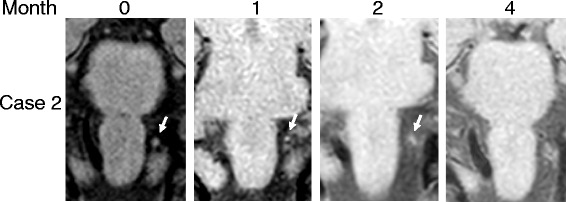
T1-VISTA imaging in Case 2. High-intensity signal areas gradually resolved and finally disappeared

A 52-year-old woman suddenly experienced vertigo and vomited while cleaning her bathtub at home and was subsequently transferred to our hospital by an ambulance. Head CT showed no abnormal findings, thus she was admitted under the suspicion of acute drug poisoning by a detergent. Although no abnormalities on a physical examination or in laboratory data were observed, her nausea and dizziness persisted. Neurologically, she presented only mild dizziness. MRI was then performed at 4 days after the onset, which showed an acute infarction in the left cerebellar hemisphere (Fig. 3a). The left VA was poorly visualized on MRA as compared to the right VA (Fig. 3b). In addition, the external diameter of the left VA on basiparallel anatomic scanning (BPAS) was narrowed, suggesting hypoplasia. The proximal portion of the left PICA was not visualized, and the adjacent distal portion appeared to be dilated on MRA (Fig. 3b). T1-VISTA showed a high-intensity signal area at the dilated portion (Fig. 3c). Conventional T1 image was obtained on admission and at 24 days after admission, but no high-intensity area was observed at both time points. Cardioembolic causes were excluded by echocardiography and 24-hour electrocardiography. According to these findings, Ischemic stroke of the cerebellum caused by dissection of the left PICA was diagnosed, and the administration of unfractionated heparin was started. Three days later, the dilated portion was enlarged on MRA, therefore, we discontinued unfractionated heparin administration. Since her blood pressure was not high, we even didn’t use antihypertensive treatment. At 8 days after the onset, cerebral angiography demonstrated the “pearl and string sign” at the proximal portion of the left PICA (Fig. 3d). Dilatation gradually normalized on MRA and the high-intensity signal area on T1-VISTA gradually reduced and disappeared at 4 months after the onset (Fig. 4).

## Conclusions

In these cases, T1-VISTA helped us for the diagnosis and follow-up of PICA-D.

PICA-D complicated with ischemic stroke is rare [4]. As an initial clinical presentation, a subarachnoid hemorrhage is the most common clinical entity following PICA-D, and a cerebral angiogram is performed early in most cases [4]. In the case of ischemic stroke, however, MRA is prioritized, and due to the inability of MRA to depict most of the normal PICA, PICA-D is likely to be overlooked. Recently, BPAS, which can visualize the surface appearance of the vertebrobasilar artery system within the cistern, has been used for the diagnosis of arterial dissection [5], however, it cannot identify the PICA in some cases. Indeed, we obtained a “pearl and string”-like sign, which reminded us of PICA-D, by the initial MRA in Case 2, while no information regarding PICA was available by MRA or BPAS in Case 1.

The BBI method clearly depicts the structure of the vessel wall by suppressing blood flow signals [6]. Since the 3D-BBI method does not require electrocardiographic triggering, it enables us to shorten the acquisition time and consequently to obtain images with thinner slices of a wider range as compared with 2D imaging. Furthermore, a cross-sectional image of any direction can be reconstructed. T1-VISTA (Phillips Inc., The Netherlands), which is similar to the 3D-BBI method, renders an intramural hematoma as a high-intensity area [3]. T1-SPACE (SIEMENS Inc., Germany) and CUBE-T1 (GE Healthcare, U.S.A.) are akin to T1-VISTA, and some groups have recently reported that these methods are useful for detecting intracranial arterial dissection [7–9]. Although cerebral angiogram findings, such as a “double lumen”, “intimal flap”, “pearl and string sign” and “string sign”, remain the golden standard for diagnosing intracranial arterial dissection, these findings are not always identified on conventional MRA. Thus, an intramural hematoma has been recognized as the definitive finding [10]. Moreover, T1-VISTA is useful in such lesions, due to its short imaging time, non-invasiveness and accuracy in identifying intramural hematomas. Regarding comparison with T1-SPACE and CUBE-T1, since the principle of T1-VISTA is the same as these methods, there might be no especial advantage of T1-VISTA over these methods.

Most of previous reports that identified an intramural hematoma using the 3D-BBI method are about VA-D [7–9]. To the best of our knowledge, only one case has been reported about the detection of an intramural hematoma by PICA-D using the 3D-BBI method [3], however, no studies about the follow-up findings have been reported. Unlike VA, PICA has many normal variations and is often not visualized on BPAS. In our two cases, it was not possible to identify the appearance of the PICA on BPAS, especially in Case 1 even on MRA, however, we suspected PICA-D due to the high-intensity area on T1-VISTA. In addition, these high-intensity signal areas gradually decreased over time, and finally disappeared in both cases. Because the vascular morphology did not change after these high-intensity signal areas disappeared (data not shown), we suspect that the vessel wall was completely repaired. No consensus has yet been formed regarding the length of time required for the high-intensity signal to disappear. The high-intensity signal areas disappeared at four or five months after the onset in our cases; therefore, we speculate that some time is required to stabilize a dissected portion. In addition, although the precise duration of antithrombotic therapy remains unknown, it may be stopped when the high-intensity signal disappears.

We performed both conventional T1 images and T1-VISTA at some time points in these cases and found that abnormal findings on T1 images mostly appeared later and smaller than those did on T1-VISTA imaging. In Case 1, conventional T1 showed no high-intensity area on admission and at 3 days later, but a pale high-intensity area was documented at the dissected lesion at 19 days after admission. Conventional T1 image was obtained on admission and at 24 days after admission in Case 2, but no high-intensity area was observed in the both time points. Compared with VA-D, the size of PICA-D is smaller, and thus the lesion would be well overlooked by T1 due to its wider slice gap. It seems to be difficult for conventional T1 to detect such a small lesion as compared with T1-VISTA.

In conclusion, T1-VISTA was able to assist in the diagnosis and follow-up of PICA-D complicated with ischemic stroke in these cases.

## Abbreviations

BBI, black blood imaging; BPAS, basiparallel anatomic scanning; CT, computed tomography; DWI, diffusion weight imaging; MRI, magnetic resonance imaging, PICA-D, Posterior inferior cerebellar dissection, VA-D, vertebral artery dissection, VISTA, volume isotropic turbo spin echo acquisition.
